# Recent Advances Regarding the Physiological Functions and Biosynthesis of D-Allulose

**DOI:** 10.3389/fmicb.2022.881037

**Published:** 2022-04-14

**Authors:** Zhou Chen, Xiao-Dong Gao, Zijie Li

**Affiliations:** Key Laboratory of Carbohydrate Chemistry and Biotechnology, Ministry of Education, School of Biotechnology, Jiangnan University, Wuxi, China

**Keywords:** rare sugars, D-allulose, D-allulose 3-epimerase, biosynthesis, aldolases, applications

## Abstract

D-Allulose, a generally regarded as safe (GRAS) sugar, is rare in nature. It is among the most promising sweeteners for future use due to its low caloric content, sucrose-like taste, and unique functions. D-Allulose has many physiological effects, such as antiobesity, antihyperglycemia, antidiabetes, anti-inflammatory, antioxidant, and neuroprotective effects. Therefore, D-allulose has important application value in the food, pharmaceutical, and healthcare industries. However, the high cost of D-allulose production limits its large-scale application. Currently, biotransformation is very attractive for D-allulose synthesis, with the two main methods of biosynthesis being the Izumoring strategy and the DHAP-dependent aldolase strategy. This article reviews recent advances regarding the physiological functions and biosynthesis of D-allulose. In addition, future perspectives on the production of D-allulose are presented.

## Introduction

Recently, the risks of obesity, hyperlipidemia, hypertension, and diabetes have increased rapidly throughout the world due to excessive intake of nutritious diets with high fat and sugar contents. Sucrose, a traditional food sweetener, plays an important role in the food industry due to its sweetness and palatability (Castro-Muñoz et al., 2022). However, sucrose has a few shortcomings, such as its high caloric value, ability to induce hyperglycemic reactions, and diabetes mellitus (Grassi et al., 2021). Therefore, low-calorie sweetener substitutes have aroused researchers’ interest (Khan et al., 2021). More than 30 kinds of rare sugars have been reported (Granström et al., 2004). They have unique biological functions and have been used as food additives, cancer cell suppressors, and building blocks for anticancer and antiviral drugs (Zhang et al., 2016b; Li et al., 2017; Guerrero-Wyss et al., 2018; Hoshikawa et al., 2018; Xia et al., 2021). D-Allulose (also called D-psicose) is well known as the most remarkable of rare sugars. It has been more than 20 years since D-allulose was first reported by Ken Izumori (Itoh et al., 1995). It exhibits 70% of the sweetness but only 0.3% of the energy deposition of sucrose. In addition, it has almost no calories (Matsuo et al., 2002). As an industrially important bioproduct, D-allulose was listed as “generally recognized as safe” (GRAS) by the US Food and Drug Administration (FDA) in 2002 and has been approved for use in candy, fruit juices, nutritional supplements, and other dietary products. Therefore, D-allulose has important application value in the food, pharmaceutical, and healthcare industries.

D-Allulose is expected to change the sweetener market because of its low caloric content and palatability, able to compete with other sugar substitutes (sugar alcohols such as xylitol, mannitol, and sorbitol). The commercial price of D-allulose is expected to approach the prices of competing sugar-substituting sweeteners, such as xylitol (US $2–5/kg), mannitol (US $1–5/kg), and sorbitol (US $1–10/kg). D-Allulose will become competitive for large-scale production in the near future, like other sweeteners consumed in the millions of tons per year. At present, the factors restricting the output and price of D-allulose include the high price of starting materials, low yields, and difficulty of isolation. In this article, recent advances regarding the physiological functions and biosynthesis of D-allulose are summarized and discussed.

## The Physiological Functions of D-Allulose

### The Effect of D-Allulose on Lipid Metabolism

D-Allulose has been reported to have antiobesity activity in animals and humans, through reductions in food intake, fat mass, and adipose tissue weight (Kimura et al., 2017; Bilal et al., 2018). In addition, several evidences suggested that D-allulose could increase energy consumption and reduce fat accumulation in normal rats (Chung et al., 2012; Ochiai et al., 2014).

Although D-allulose can decrease the weight of adipose tissue, the mechanism remains unknown. It can decrease the fatty acid synthase (FSA) activity and increase β-oxidation and carnitine palmitoyltransferase (CPT) activity in epididymal white adipose tissue (WAT; Han et al., 2016). Furthermore, dietary D-allulose can suppress the expression of lipogenesis-related acetyl-CoA carboxylase alpha (ACCα) in epididymal WAT. In addition, dietary D-allulose can stimulate the expression of fatty-acid-oxidation-related AMP-activated protein kinase alpha 2 (AMPKα2), hormone-sensitive lipase (HSL), and peroxisome proliferator activated receptor alpha (PPARα; Chen et al., 2019). Therefore, D-allulose has potential antiobesity properties.

### The Antihyperglycemic Effect of D-Allulose

Adequate nutrition has led to rapidly increasing incidences of obesity and obesity-induced type 2 diabetes mellitus (T2DM) around the world, significantly increasing the costs of treating these chronic diseases. Therefore, it is very important to identify effective therapeutic interventions for the treatment of diabetes and its complications. D-Allulose has attracted much attention because of its promising antihyperglycemic properties, being able to control plasma glucose level, body weight, and fat mass (Matsuo and Izumori, 2009; Hossain et al., 2015; Lee et al., 2020). However, the mechanism of its antidiabetes effects remains unclear. It has been speculated that D-allulose affects the levels of blood glucose and insulin secretion or the activities of glucosidase and lipolytic enzymes. D-Allulose has been shown to significantly suppress the increase in plasma glucose concentration induced by sucrose or maltose (Matsuo and Izumori, 2009). In addition, D-allulose has been found to potently inhibit the activities of intestinal sucrase and maltase. The addition of D-allulose (5 g) can significantly suppress the increase in blood glucose induced by oral maltodextrin (75 g) in normal adults. Additionally, oral D-allulose alone does not affect the blood levels of glucose and insulin (Iida et al., 2008). D-Allulose has also been found to inhibit the increase in postprandial blood glucose level, mainly in patients with borderline diabetes, with no side effects or clinical problems observed following 12 weeks of continuous D-allulose intake (Hayashi et al., 2010).

Although several investigations have revealed that D-allulose has antidiabetes activity, the mechanism has yet to be fully elucidated. D-Glucose metabolism in the liver is regulated by the nucleocytoplasmic shift of glucokinase (Hossain et al., 2015). At low glucose levels, glucokinase maintains inactive forms by binding with glucokinase regulatory protein (GKRP), which is recruited in the hepatocyte nucleus. When glucose reaches a high level, glucokinase is activated by separation from the glucokinase-GKRP complex and is translocated from the nucleus to the cytoplasm, where it participates in glycogen metabolism and blood glucose homeostasis (Liu et al., 2012). For example, activation of glucokinase can improve glucose tolerance and insulin sensitivity (Shintani et al., 2017b). Impaired functioning of hepatic glucokinase results in the pathogenesis of hyperglycemia in diabetes (Basu et al., 2001). Therefore, glucokinase is considered a candidate target for antidiabetes drugs (Lloyd et al., 2013). In addition, glucokinase is activated by a variety of fructose phosphates, such as fructose 6-phosphate (F6P) and fructose 1-phosphate (F1P; Pfefferkorn, 2013). D-Allulose 1-phosphate, similar to fructose-1-phosphate, also activates glucokinase. Therefore, D-allulose can increase the utilization of hepatic glucose (Toyoda et al., 2010). Glucose-6-phosphatase (G6Pase) contributes to hyperglycemia in diabetes mellitus and regulates the rate-limiting steps in hepatic gluconeogenic flux (Herling et al., 1998). D-Allulose can regulate blood glucose level and exert hypolipidemic effects by decreasing the activity of G6Pase (Nagata et al., 2015). It has been reported that oral administration of D-allulose can stimulate GLP-1 secretion and thus be used to prevent and treat glucose intolerance (Hayakawa et al., 2018). Recently, D-allulose was confirmed to induce GLP-1 release, activate vagal afferent signaling, reduce feeding, and restrict hyperglycemia in healthy and obese diabetic rats. Furthermore, oral D-allulose can correct arrhythmic overeating, obesity, and diabetes (Iwasaki et al., 2018).

### Other Physiological Functions of D-Allulose

**Figure 1:**
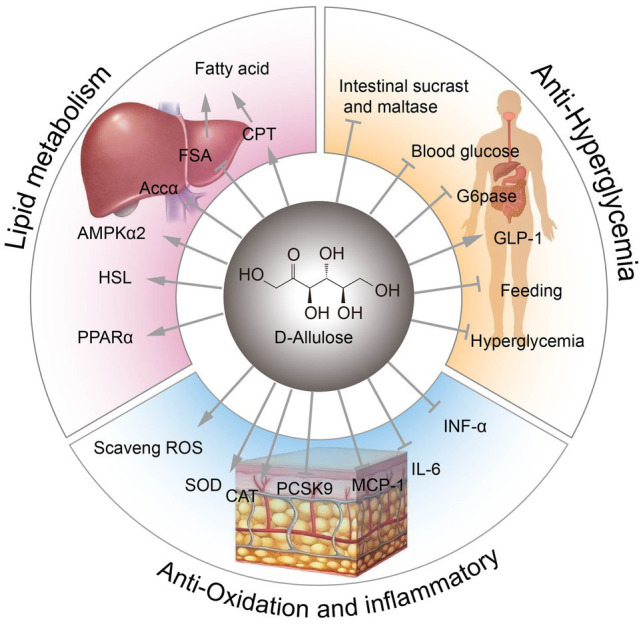
The physiological functions of D-allulose. FSA, fatty acid synthase; CPT, carnitine palmitoyltransferase; ACCα, acetyl-CoA carboxylase alpha; AMPKα, AMP-activated protein kinase alpha; HSL, hormone-sensitive lipase; PPARα, peroxisome proliferator activated receptor alpha; ROS, reactive oxygen species; SOD, superoxide dismutase; CAT, catalase activity; Pcsk9, proprotein convertase subtilisin/kexin type 9; MCP-1, monocyte chemoattractant protein 1; G6Pase, glucose-6-phosphatase.

D-Allulose exhibits anti-inflammatory effects by suppressing serum levels of proinflammatory cytokines, such as tumor necrosis factor-alpha (TNF-α), interleukin 6 (IL-6), and monocyte chemoattractant protein 1 (MCP-1). These cytokines are derived mainly from visceral adipose tissues (Moller and Berger, 2003; Kim et al., 2017). D-Allulose also has antioxidant effects, scavenging reactive oxygen species (ROS) to protect 6-hydroxydopamine-induced apoptosis or prevent testicular injury (Takata et al., 2005; Suna et al., 2007). D-Allulose can also extend lifespan by increasing superoxide dismutase (SOD) activity and catalase (CAT) activity (Shintani et al., 2017a). Moreover, D-allulose has been shown to alter serum cholesterol levels in hamsters, in part by reducing proprotein convertase subtilisin/kexin type 9 (Pcsk9) level (Kanasaki et al., 2019). Furthermore, D-allulose could improve systemic and muscle insulin sensitivity in conscious rats (Natsume et al., 2021). Various physiological functions of D-allulose are illustrated in Figure 1.

## Biological Production of D-Allulose

As described above, D-allulose has many useful physiological functions. However, D-allulose is rare in nature, which greatly restricts its large-scale application. Traditional chemical synthesis of D-allulose usually involves tedious reactions and many side reactions (McDonald, 1967; Doner, 1979). It is difficult to obtain a single configuration of product using chemical methods. In contrast, bioconversion approaches have many advantages, including mild reaction conditions, few byproducts, simple purification steps, and environmentally friendly properties (Zhang et al., 2021). Therefore, biotransformation has gradually become the main method of D-allulose synthesis. At present, the biological preparation of D-allulose is achieved mainly *via* two strategies: (1) the Izumoring strategy and (2) the DHAP-dependent aldolase strategy.

### D-Allulose Production Using the Izumoring Strategy

#### D-Allulose Production From D-Fructose Using D-Tagatose 3-Epimerase Family Enzymes

**Figure 2:**
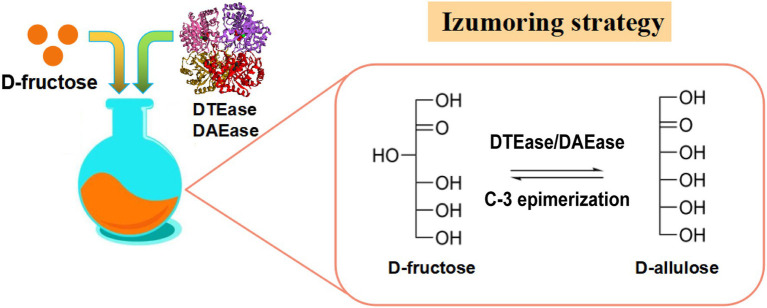
Izumoring strategy for production of D-allulose. DTEase, D-tagatose 3-epimerase; DAEase, D-allulose 3-epimerase.

The Izumoring strategy is a promising approach for the bioproduction of any kind of hexose sugar and involves D-tagatose 3-epimerases (DTEases), polyol dehydrogenases, and aldose isomerases (Izumori, 2006). In the past few decades, the Izumoring strategy has proven effective for biosynthesizing rare sugars. DTEase family enzymes are the key enzymes for the biosynthesis of D-allulose from D-fructose (Figure 2) and include DTEase and D-allulose 3-epimerase (DAEase). DAEase exhibits higher specificity for D-allulose than D-tagatose (Kim et al., 2006). Since the first DTEase from *Pseudomonas cichorii* ST-24 was identified in 1993, other DTEase enzymes were isolated from a variety of species, such as *Agrobacterium tumefaciens* (Kim et al., 2006), *Clostridium Bolteae* (Jia et al., 2014), *Dorea* sp. CAG317 (Zhang et al., 2015), *Ruminococcus* sp. 5_1_39BFAA (Chen et al., 2016), *Treponema primitia* ZAS-1 (Zhang et al., 2016c), *Rhodobacter sphaeroides* (Qi et al., 2017), *Ruminococcus* sp. (Li et al., 2018), *Arthrobacter globiformis* M30 (Yoshihara et al., 2017), *Clostridium cellulolyticum* H10 (Su et al., 2018), *Sinorhizobium* sp. (Zhu et al., 2019b), and *Rhodopirellula baltica* SH 1 (Zhang et al., 2020). Details of the catalytic properties of DTEase-family enzymes from different species are presented in Table 1. Itoh et al. (1995) first reported the immobilization of DTEase from *P. cichorii* on chitopearl beads and 90 g D-allulose from 500 g D-fructose was generated. Since then, the methods for DTEase immobilization have attracted extensive interest (Lim et al., 2009; Tseng et al., 2014; Narayan Patel et al., 2018), and the immobilized enzymes exhibited improved thermal stability and storage stability.

**Table 1:** Properties of DTEase family enzymes.

Ketose 3-Epimerases	Enzyme source	pH	Temperature (°C)	Metal ion	Conversion (%)
DAEase (Jia et al., 2014)	*Clostridium Bolteae*	7.0	55	Co^2+^	29%
DAEase (Zhang et al., 2015)	*Dorea* sp. *CAG317*	6.0	70	Co^2+^	30%
DAEase (Chen et al., 2016)	*Ruminococcus* sp. *5_1_39BFAA*	8.0	55	Mn^2+^	29%
DAEase (Zhang et al., 2016c)	Treponema primitiaZAS-1	8.0	70	Co^2+^	28%
DTEase (Li et al., 2017)	*Ruminococcus* sp.	7.5–8.0	60	Mn^2+^	26%
DAEase (Yoshihara et al., 2017)	*Arthrobacter globiformis M30*	7.0–8.0	70	Mg^2+^	24%
DAEase (Su et al., 2018)	*Clostridium cellulolyticum H10*	6.5–8.5	65	Ca^2+^	28%
DTEase (Zhu et al., 2019b)	*Sinorhizobium* sp.	8.0	50	Mn^2+^	42.5%
DTEase (Zhang et al., 2020)	*Rhodopirellula baltica* SH 1	7.0	35	Mn^2+^	25.86%

#### Biosynthesis of D-Allulose From Inexpensive Materials Based on DTEase Enzymes

Currently, agricultural byproducts, such as fruit and vegetable residue, are generating major agricultural problems (Lai et al., 2017). Agricultural residues are usually buried in landfills or incinerated (Park and Yoon, 2015). However, these residues contain large amounts of dietary fibers and sugars, including sucrose, D-glucose, and D-fructose. The conversion of dietary fiber and sugars to high value-added products represents a significant step towards alleviating agricultural problems.

**Figure 3:**
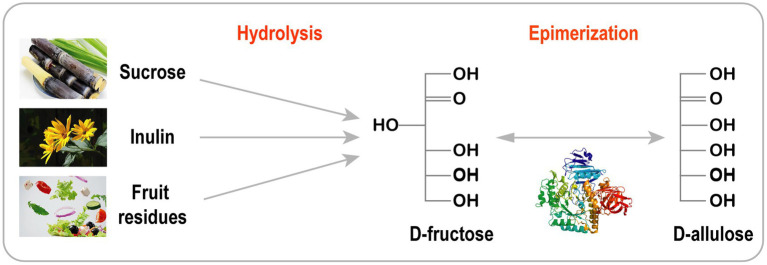
Biosynthesis of D-allulose from inexpensive raw material.

Cascade catalysis is considered as a very attractive approach compared with traditional step-by-step synthesis. This strategy is often used to produce rare sugars from inexpensive materials, such as sucrose, Jerusalem artichoke, inulin, and fruit/vegetable residues (Figure 3; Wagner et al., 2015; Song et al., 2016, 2017; Zhang et al., 2017; Yang et al., 2019; Li et al., 2020a). D-Allulose has been efficiently synthesized from sucrose using purified recombinant invertase, D-xylose isomerase, and DTEase. Moreover, practical integration of a cascade solution with simulated moving bed (SMB) chromatography has been used to produce pure D-allulose (99.9%) with very high yields (89%; Wagner et al., 2015). D-Allulose can also be synthesized in a two-step cascade reaction involving Jerusalem artichoke hydrolysis (Song et al., 2017), cruciferous vegetable residue (Song et al., 2016) and inulin (Li et al., 2020a). To decrease production costs and avoid enzyme purification, Zhu et al. developed a one-pot two-enzyme reaction system with a novel exo-inulinase from *Bacillus velezensis* (BvInu) and DAEase from *Ruminococcus* sp. for the production of D-allulose from Jerusalem artichoke. BvInu and DAEase were expressed in *Bacillus subtilis* and secreted into supernatant without purification. Under the optimal ratio of BvInu/RDAE (80:40 U/g inulin) at 50°C for 2 h, 10.3 g/L of D-allulose was obtained from 50 g/L inulin (Zhu et al., 2020).

#### Biosynthesis of D-Allulose Using Microorganisms

**Figure 4:**
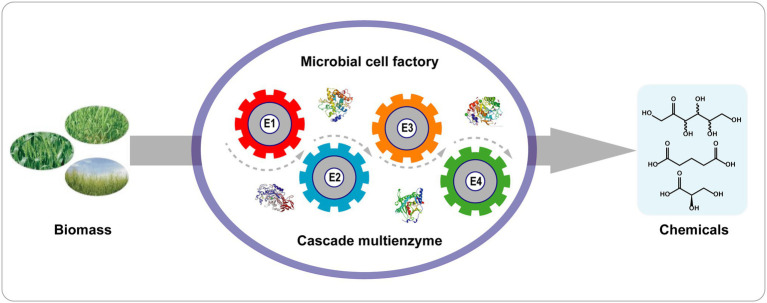
Cascade biotransformation with a single recombinant cell. E1–E4 represent enzyme 1–4.

Compared with one-pot cascade reactions, whole-cell biocatalyst reactions have several advantages (Figure 4): (1) Cells containing enzymes are easily obtained without tedious purification of enzymes; (2) the cellular context provides a suitable microenvironment and cofactor regeneration (ATP, NAD^+^/NADH); (3) the cell walls and membranes protect the enzymes against harsh reaction conditions; and (4) the colocalization of multiple enzymes within the cell enhances the local concentrations of enzymes and decreases the diffusion of intermediates in cascade reactions (Wu and Li, 2018).

Engineered *Escherichia coli* is one of the most commonly used organisms for the production of D-allulose because of its clear background, fast growth rates, simple culture, and stable genetics. DAEase and D-glucose isomerase (GIase) from *Acidothermus cellulolyticus* were coexpressed to produce D-allulose from D-glucose (Zhang et al., 2017). Similarly, DAEase and xylose isomerase (XI) were coexpressed to produce D-allulose with D-glucose as the substrate (Chen et al., 2017). In the above two approaches, D-glucose was first converted to D-fructose by GIase or XI, and then, D-fructose was immediately isomerized to D-allulose by DAEase.

Although good productivity was obtained by engineering *E. coli*, which was not applicable in the food industry because of the endotoxins and their non-food grade classification. To date, DAEase has been successfully expressed in several food-safe strains, such as *B. subtilis*, *Saccharomyces cerevisiae*, and *Corynebacterium glutamicum* (Li et al., 2015b; He et al., 2016). He et al. (2016) displayed DTEase from *Clostridium scindens* ATCC 35704 on the surface of *B. subtilis* spores for the production of D-allulose from D-fructose. DAEase was fused at the C-terminus of CotZ and exhibited high thermostability. After five cycles of utilization, 60% activity was maintained (He et al., 2016). In our previous study, we innovated a spore surface display technique to produce D-allulose from D-glucose. In this approach, the key enzymes XI from *Thermus thermophilus* and DAEase from *A. tumefaciens* were immobilized on *S. cerevisiae* spores using biological and chemical methods, respectively (Li et al., 2015b). In addition, multiple DAEases and invertase (INV) were overexpressed in *C. glutamicum*, and the engineered cells immobilized with alginate were subjected to a cascade reaction in a one-pot, two-step reaction system to generate D-allulose from cane molasses. After reaction for 8 h, 61.2 g/L D-allulose was obtained, which represented 17.4% of the total monosaccharides (Yang et al., 2019). Currently, the Izumoring strategy is the main method for industrial production of D-allulose, which could be achieved by using SMB. Additionally, the separated D-fructose can be reused for D-allulose production. In brief, Izumoring strategy is the most simple and common way to biosynthesize D-allulose. However, the limitation of thermodynamic balance is a bottleneck which restricts the large-scale application of D-allulose in the food industry.

### D-Allulose Production Using DHAP-Dependent Aldolases

As mentioned above, various approaches based on the Izumoring strategy have been developed for D-allulose production from inexpensive starting materials, such as Jerusalem artichoke, inulin, and agricultural residues. However, the reaction catalyzed by the key enzyme DAEase is reversible, and the conversion rate is low, which leads to a high price for the resulting D-allulose. Moreover, most strains used in the synthesis of D-allulose are not GRAS microorganisms. Therefore, it is extremely important to construct a cost-efficient and safe platform for the mass production of D-allulose.

**Figure 5:**
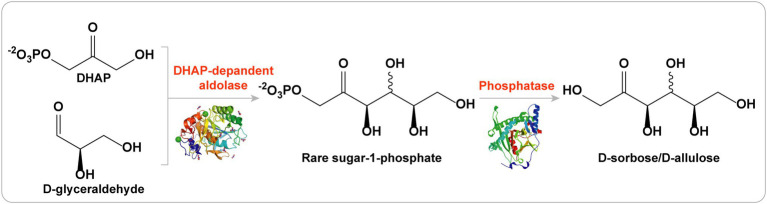
DHAP-dependent aldolases strategy for the production of D-allulose.

Stereoselective aldol additions catalyzed by aldolases have become an essential tool for C-C asymmetric synthesis (Iturrate et al., 2010). Among the members of the aldolase family, DHAP-dependent aldolases are the most widely used for the synthesis of many carbohydrate compounds that are difficult to synthesize by traditional chemical methods (Bosshart et al., 2013). There are four types of DHAP-dependent aldolases: L-rhamnulose-1-phosphate aldolase (RhaD), L-fuculose-1-phosphate aldolase (FucA), D-fructose-1,6-bisphosphate aldolase (FruA), and D-tagatose-1,6-bisphosphate aldolase (TagA). Stereoselective aldol reactions of these four aldolases are complementary to each other. In theory, a set of four ketoses can be obtained by using DHAP as the donor and the same aldehyde as the receptor (Gustavo, 2000). Therefore, DHAP-dependent aldolases are highly suitable for the synthesis of various rare sugars (including D-allulose, D-sorbose, D-tagatose, and L-fructose) due to their unique stereoselectivities (Figure 5; Brovetto et al., 2011; Dai et al., 2021).

#### Biosynthesis of D-Allulose Using RhaD Aldolase With D-Glyceraldehyde as the Acceptor

**Figure 6:**
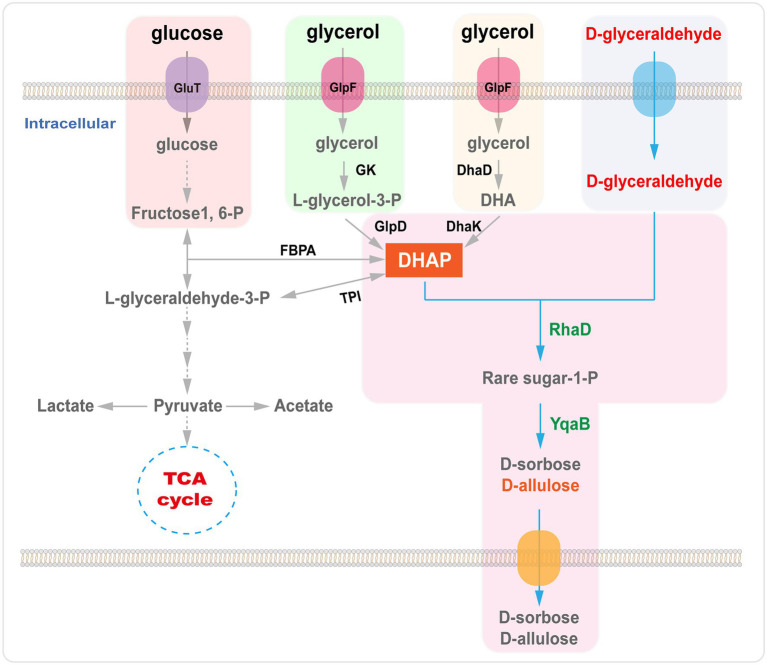
Biosynthesis of D-allulose using RhaD aldolase from glycerol or glucose. GlpF, glycerol transporter; GK, glycerol kinase; GlpD, glycerol 3-phosphate dehydrogenase; DhaD, glycerol dehydrogenase; DhaK, dihydroxyacetone kinases; FBPA, fructose 1, 6-diphosphate aldolase; TPI, triose phosphate isomerase; RhaD, L-rhamnulose-1-phosphate aldolase; YqaB, fructose-1-phosphatase.

A main disadvantage of DHAP-dependent aldolase strategies is that the donor substrate DHAP is very expensive and unstable for large-scale synthesis (Schümperli et al., 2007). However, there are several routes for DHAP synthesis *via* enzymatic strategies. For instance, DHAP can be obtained *via* dihydroxyacetone (DHA), glycerol or glycerol 3-phosphate or through metabolic pathways from an inexpensive raw material, such as glucose or glycerol (Figure 6; Sánchez-Moreno et al., 2004; Li et al., 2012, 2015a; Wei et al., 2015; Yang et al., 2015, 2016). In engineered *E*. *coli*, catalysis of the aldol reaction by RhaD to synthesize rare sugars (D-allulose and D-sorbose) has been achieved using glucose as the carbon source and a continuous supply of D-glyceraldehyde into the medium. Following optimization of fermentative conditions, the isolated yield of D-allulose and D-sorbose was 0.21 mol/mol D-glyceraldehyde (Wei et al., 2015). D-Allulose and D-sorbose have been produced in a GRAS *C. glutamicum* strain with glucose and D-glyceraldehyde as feedstocks. The recombinant *C. glutamicum* strains harboring RhaD and fructose-1-phosphatase (YqaB) have been found to accumulate 19.5 g/L of D-sorbose and 13.4 g/L of D-allulose in a fed batch fermentation (Yang et al., 2015). In addition, D-allulose and D-sorbose have been produced in a recombinant *E. coli* strain overexpressing aldolase RhaD and YqaB from glycerol by fermentation. After 15 h of fermentation, the concentrations of D-sorbose (1.6 g/L) and D-allulose (1.23 g/L) were determined in the supernatant (Li et al., 2015a). Recently, our group constructed an efficient system for whole-cell cascade synthesis of D-sorbose and D-allulose from glycerol and D-glyceraldehyde, which yielded 15.3 g/L of D-sorbose and 6.4 g/L of D-allulose in a batch biotransformation (Chen et al., 2020a).

#### Biosynthesis of D-Allulose From Glycerol as the Sole Substrate

A microorganism platform for D-allulose synthesis based on aldolase RhaD would hold much promise for large-scale production. The main challenge is the high cost of the donor substrate DHAP and acceptor molecule D-glyceraldehyde. Fortunately, the problem of how to accumulate DHAP using the inexpensive industrial byproduct glycerol as a “green” carbon source has been solved. Thus, the next issue is to identify a novel enzyme to convert low-value glycerol to D-glyceraldehyde. A glycerol dehydrogenase or glycerol oxidase that can efficiently catalyze glycerol to D-glyceraldehyde is needed.

**Figure 7:**
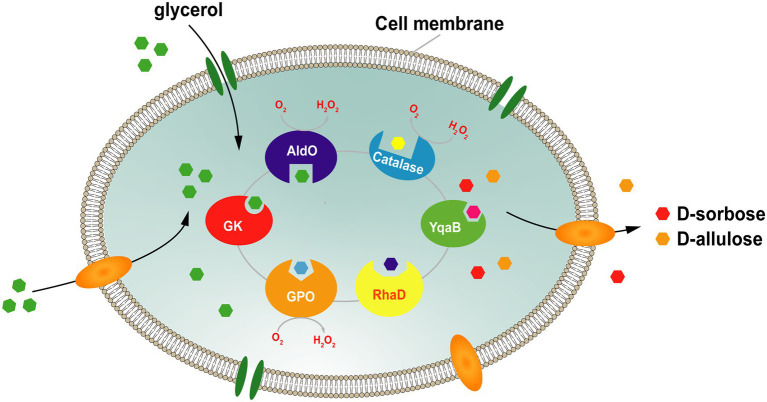
Strategy for the whole-cell synthesis of D-sorbose and D-allulose from glycerol. GK, glycerol kinase; GPO, glycerol oxidase; AldO; alditol oxidase; RhaD, L-rhamnulose-1-phosphate aldolase; YqaB, fructose-1-phosphatase.

To address this issue, our group constructed a platform for the whole-cell cascade synthesis of D-sorbose and D-allulose from glycerol as the sole substrate. In this system (Figure 7), the donor substrate DHAP is generated by the glycerol assimilation pathway, and the endogenous DHAP is produced *via* overexpression of glycerol kinase (GK) and glycerol phosphate oxidase (GPO). The acceptor D-glyceraldehyde is directly generated from glycerol by alditol oxidase from *Streptomyces coelicolor* A3 (AldO*_S.coe_*). Then, RhaD catalyzes the aldol reaction between DHAP and D-glyceraldehyde to form the corresponding ketose-1-phosphate. Finally, D-sorbose and D-allulose are obtained by removing the phosphate group by YqaB phosphatase. Using this system, the production yields of D-sorbose and D-allulose were enhanced approximately 1.7-fold and 1.2-fold after the overexpression of peroxidase (Prx02 or KatE) to eliminate the harmful effects of H_2_O_2_. A total of 7.9 g/L of D-sorbose and D-allulose was obtained from glycerol, with a total conversion rate of 17.7% (Chen et al., 2020b).

**Figure 8:**
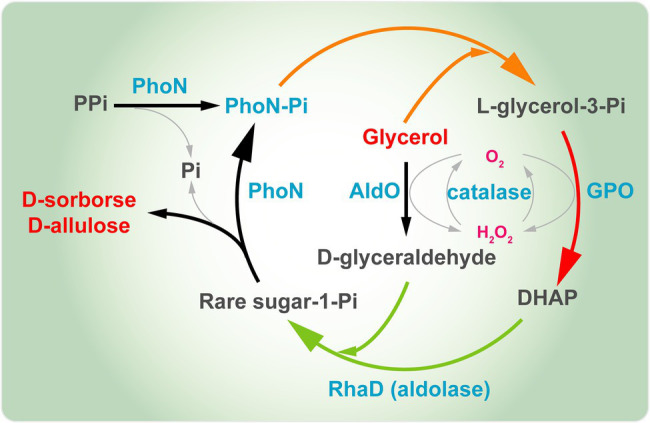
Multienzyme synthesis of D-sorbose and D-allulose from glycerol. PhoN, acid phosphatase; GPO, glycerol oxidase; AldO; alditol oxidase; RhaD, L-rhamnulose-1-phosphate aldolase; DHAP, dihydroxyacetone phosphate.

Our group also constructed a one-pot multienzyme system for the synthesis of D-sorbose and D-allulose from glycerol as the sole carbon source (Figure 8). Here, acid phosphatase PhoN from *Shigella flexneri* (PhoN-Sf) was introduced to the system instead of GK and YqaB, which catalyzed the phosphorylation reaction of glycerol in the first step and helped recycle the phosphate of ketose-1-phosphate in the last step to provide free rare-sugar molecules. AldO*_S.coe_* was introduced to the above multienzyme cascade to synthesize D-sorbose and D-allulose exclusively from the readily available glycerol. Finally, 14.8 g/L of D-sorbose and D-allulose was obtained from glycerol (Li et al., 2020b). The above strategy also represents a very useful and low-cost approach for producing various other rare sugars. In a word, biosynthesis of D-allulose by fermentation based on DHAP-dependent aldolases is very promising. It would be more profitable to construct a cell factory based on DHAP-dependent aldolases strategy for the synthesis of D-allulose.

### Thermodynamics-Driven Production of D-Allulose Based on Phosphorylation–Dephosphorylation Strategy

**Figure 9:**
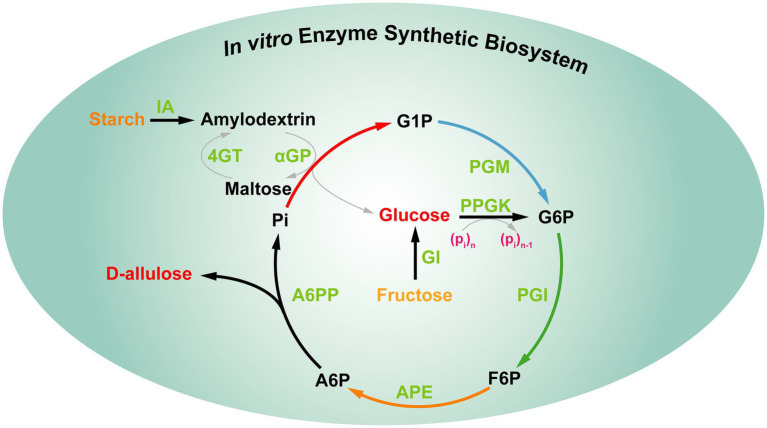
*In vitro* multi-enzyme synthesis of D-allulose from starch. IA, isoamylase; 4GT, 4-α-glucanotransferase; αGP, α-glucan phosphorylase; PGM, phosphoglucomutase; PPGK, polyphosphate glucokinase; PGI, phosphoglucose isomerase; GI, glucose isomerase; APE, D-allulose 6-phosphate 3-epimerase; A6PP, D-allulose 6-phosphate phosphatase; G1P, glucose-1-phosphate; G6P, glucose-6-phosphate; F6P, fructose-6-phosphate; A6P, D-allulose-6-phosphate.

In fact, the above strategies still cannot meet the food industrial demand of D-allulose. Therefore, it is interesting to develop a novel strategy for D-allulose production with highly efficient and low-cost green biomanufacturing. Recently, You et al. constructed an *in vitro* synthetic enzymatic biosystem for D-allulose from inexpensive starch based on “the thermodynamic-driven strategy” (Figure 9). This *in vitro* biosystem involved five core enzymes, the reactions occur as follows: (1) maltodextrin (a derivative of starch) was phosphorylated to generate glucose-1-phosphate (G1P) by α-glucan phosphorylase (αGP) with phosphate as co-substrate. (2) G1P was converted to glucose-6-phosphate (G6P) catalyzed by phosphoglucomutase (PGM). (3) G6P was converted to fructose-6-phosphate (F6P) catalyzed by phosphoglucose isomerase (PGI). (4) F6P was epimerized to generate D-allulose-6-phosphate (A6P) catalyzed by D-allulose 6-phosphate 3-epimerase (A6PE). (5) A6P was dephosphorylated to generate D-allulose and phosphate catalyzed by D-allulose-6-phosphate phosphatase (A6PP). Besides, the other four auxiliary enzymes [isoamylase (IA), 4-α-glucanotransferase (4GT), polyphosphate glucokinase (PPGK), and glucose isomerase (GI)] were added the reaction system at different timepoints to achieve the complete utilization of maltodextrin for D-allulose production. In this biosystem, the Gibbs energy of A6P dephosphorylation to D-allulose is −15.5 kJ/mol, which is irreversible, indicating that the dephosphorylation step for D-allulose production is thermodynamically favorable and unidirectional to push the overall reaction toward completeness. After the optimization of the reaction conditions, the production yields of D-allulose from 10 and 50 g/L starch reached 88.2 and 79.2%, respectively (Li et al., 2021). All in all, this thermodynamics-driven strategy provides a promising alternative for the cost-efficient production of D-allulose. Due to the system involves multiple enzymes and the steps of enzyme purification are cumbersome, the above strategy still cannot meet the needs of industrialization.

## Summary and Future Perspectives

The most common method for D-allulose production is based on the Izumoring strategy, which is limited by thermodynamic equilibrium resulting in a low conversion rate and relatively high synthetic cost. Therefore, novel and robust DTEase-family enzymes need to be discovered. Moreover, the strategy of direction evolution (Zhang et al., 2016a; Zhu et al., 2019a) or enzyme immobilization (Ran et al., 2019; Wong et al., 2020) to improve the catalytic performance of DTEase will be very useful. For DHAP-dependent aldolase strategy, D-allulose and D-sorbose are generated simultaneously by RhaD with D-glyceraldehyde as the acceptor. To improve the stereoselectivity of aldolases, various advanced techniques and methods, including directed evolution (d’Oelsnitz and Ellington, 2018; Shepelin et al., 2018; Currin et al., 2021), high-throughput screening techniques (Ung et al., 2018; Rienzo et al., 2021), and rational engineering (Damborsky and Brezovsky, 2014; Windle et al., 2014; Kim et al., 2020), could be adopted. Obviously, thermodynamics-driven strategy is a promising method for D-allulose production. Construction of microbial cell factory using this approach would be a direction in the future. A variety of metabolic tools will contribute to the industrial production of D-allulose, such as CRISPR/Cas9 (Wu et al., 2019; Nishida and Kondo, 2021), self-assembly (Liu et al., 2019; Lange and Polizzi, 2021), and dynamic regulation (Hartline et al., 2021; Zhu et al., 2021). Hopefully, D-allulose will become affordable to ordinary consumers in the near future.

## Author Contributions

ZC performed data curation and writing—original draft preparation. X-DG was involved in visualization, investigation, and supervision. ZL performed writing—reviewing and editing. All authors contributed to the article and approved the submitted version.

## Funding

This work was supported by the National Natural Science Foundation of China (nos. 32171475, 31971216), the China Postdoctoral Science Foundation (no. 2021M691285), Shandong Provincial Major Scientific and Technological Innovation Project (no. 2019JZZY011006), Natural Science Foundation of Jiangsu Province (no. BK20210465), and Program of Introducing Talents of Discipline to Universities (no. 111-2-06).

## Conflict of Interest

The authors declare that the research was conducted in the absence of any commercial or financial relationships that could be construed as a potential conflict of interest.

## Publisher’s Note

All claims expressed in this article are solely those of the authors and do not necessarily represent those of their affiliated organizations, or those of the publisher, the editors and the reviewers. Any product that may be evaluated in this article, or claim that may be made by its manufacturer, is not guaranteed or endorsed by the publisher.
